# AI-Assisted X-ray Fracture Detection in Residency Training: Evaluation in Pediatric and Adult Trauma Patients

**DOI:** 10.3390/diagnostics14060596

**Published:** 2024-03-11

**Authors:** Mathias Meetschen, Luca Salhöfer, Nikolas Beck, Lennard Kroll, Christoph David Ziegenfuß, Benedikt Michael Schaarschmidt, Michael Forsting, Shamoun Mizan, Lale Umutlu, René Hosch, Felix Nensa, Johannes Haubold

**Affiliations:** 1 Department of Diagnostic and Interventional Radiology and Neuroradiology, University Hospital Essen, 45147 Essen, Germany; 2 Institute for Artificial Intelligence in Medicine, University Hospital Essen, 45131 Essen, Germany

**Keywords:** X-rays, fractures, bone, artificial intelligence, diagnostic imaging, quality improvement

## Abstract

**Background**: This study aimed to evaluate the impact of an AI-assisted fracture detection program on radiology residents’ performance in pediatric and adult trauma patients and assess its implications for residency training. **Methods**: This study, conducted retrospectively, included 200 radiographs from participants aged 1 to 95 years (mean age: 40.7 ± 24.5 years), encompassing various body regions. Among these, 50% (100/200) displayed at least one fracture, totaling one hundred thirty-five fractures, assessed by four radiology residents with different experience levels. A machine learning algorithm was employed for fracture detection, and the ground truth was established by consensus among two experienced senior radiologists. Fracture detection accuracy, reporting time, and confidence were evaluated with and without AI support. **Results**: Radiology residents’ sensitivity for fracture detection improved significantly with AI support (58% without AI vs. 77% with AI, *p* < 0.001), while specificity showed minor improvements (77% without AI vs. 79% with AI, *p* = 0.0653). AI stand-alone performance achieved a sensitivity of 93% with a specificity of 77%. AI support for fracture detection significantly reduced interpretation time for radiology residents by an average of approximately 2.6 s (*p* = 0.0156) and increased resident confidence in the findings (*p* = 0.0013). **Conclusion**: AI support significantly enhanced fracture detection sensitivity among radiology residents, particularly benefiting less experienced radiologists. It does not compromise specificity and reduces interpretation time, contributing to improved efficiency. This study underscores AI’s potential in radiology, emphasizing its role in training and interpretation improvement.

## 1. Introduction

The number of patients presenting to emergency departments is experiencing a persistent upward trend [1]. Radiographs are commonly used to diagnose trauma-related injuries [2,3]. With its wide availability, low radiation exposure, and low costs, radiography offers distinct advantages in the initial diagnosis of fractures in the emergency department [1,4]. Reporting of radiographs is often delayed due to increased workload or results in diagnostic errors due to missed fractures [3,5]. In addition, the interpretation of radiographs is often assigned to experienced residents [3]. Accurate interpretation of radiographs requires a substantial level of expertise in the field of radiology. However, radiology residents, who are still in the early stages of their training, have limited experience. Consequently, they encounter challenges and uncertainties when interpreting radiographs, which can potentially result in misdiagnosis. The lack of extensive experience among radiology residents, in particular, contributes to the increased likelihood of errors and inaccuracies in reading radiographs. Hence, providing assistance to radiology residents in interpreting radiographs outside of normal working hours, when support from senior radiologists is sparse, becomes even more crucial. In the past, a number of different AI-based programs have been tested to assist in the diagnosis of fractures in radiographs of the extremities. Previous studies have already demonstrated enhanced sensitivity in fracture detection using AI-based programs. However, these studies have predominantly focused on single body regions, particularly extremities [3,4,6,7,8,9,10,11,12,13,14,15,16]. In the daily clinical setting of the emergency department, radiologists are confronted with a variety of radiographs from different body regions, not just those of extremities. This study distinguishes itself by adequately representing the wide array of radiographs in the clinical setting of the emergency department, thus providing a realistic picture of the use of AI in fracture detection across a broad cross-section of body regions. This comprehensive approach highlights the novel contribution of our work to the field, expanding the application of AI beyond the limitations of previous studies focused on specific body parts. 

This study aimed to evaluate the impact of an AI-assisted fracture detection program as a tool for radiology residents in pediatric and adult trauma patients to improve sensitivity, specificity, and reading speed. The objective was to investigate the extent to which assistance from AI-based programs can enhance diagnostic accuracy in fracture detection and reduce radiology residents’ uncertainty, thus improving feelings of safety in fracture detection.

## 2. Materials and Methods

### 2.1. Study Population and Design

**Figure 1:**
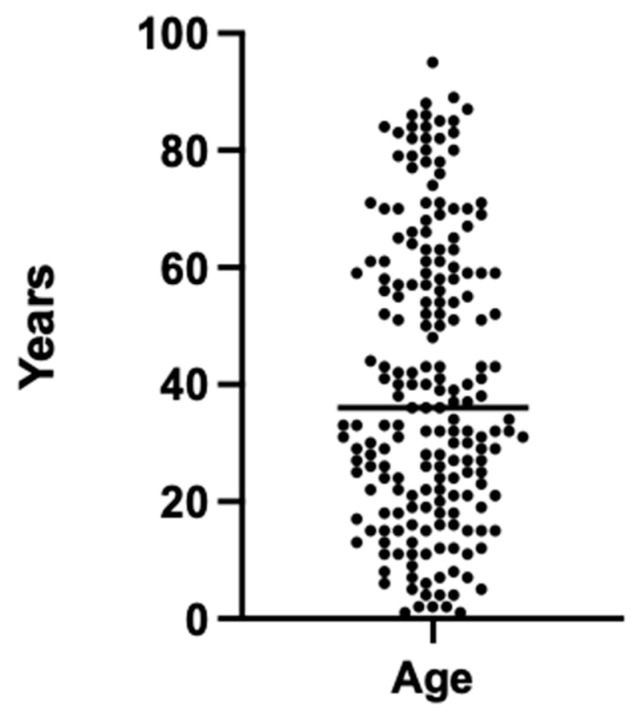
Age distribution of the patient cohort for the 200 radiographic images used.

The study cohort consisted of a total of 200 radiographic examinations involving 95 female and 105 male participants. The participants ranged in age from 1 to 95 years, with a mean age of 40.7 ± 24.5 years. The sample included individuals across a wide age spectrum, encompassing both pediatric and adult populations (Figure 1).

**Figure 2:**
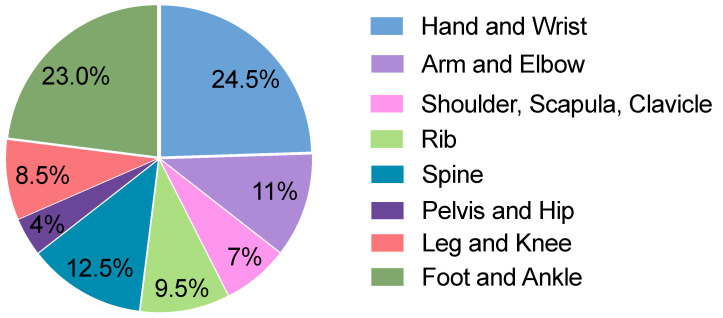
Distribution of radiographic images in the cohort across different body regions.

A single-center in-house search was conducted for conventional radiographic images. A total of 100 radiographic images with at least one fracture and 100 images without a fracture were randomly selected from July 2022 to August 2022. Among the 100 images with 1 or more fractures, a total of 135 fractures were identified. The body regions investigated covered a wide range of the body, including the hand, wrist, arm, elbow, shoulder, scapula, clavicle, ribs, spine, pelvis, hip joints, legs, knees, ankles, and feet. The frequency of different body regions is presented in Figure 2. The frequency of radiographs of various body regions of patients with a wide range of ages is based on the frequency of occurrence in the emergency department of the examining hospital, which allows a wide range of findings to be analyzed.

After assembling the collective, the radiographic images were evaluated by four radiology residents with different levels of training in the radiology residency program, ranging from 4.5 to 24.5 months of experience. The residents evaluated the 200 radiographs according to various criteria, including the presence of fractures, the number of fractures, the bones involved, the time required for diagnosis, and the degree of certainty of the diagnosis.

To assess the confidence levels of the residents, a 6-point Likert scale was used, with responses ranging from 1 (representing ‘I feel very confident’) to 6 (representing ‘I feel very uncertain’). Half of the images were shown with AI support, and the other half without AI support. To exclude bias, two residents were shown 100 images with and 100 images without AI support, and the other two residents were shown exactly the opposite. 

### 2.2. Ground Truth

The radiographs were reviewed by two consultant radiologists with 7 and 10 years of experience in musculoskeletal imaging. Consensus-based decisions were made during the evaluation process. In cases of indeterminacy, supplementary imaging modalities, such as magnetic resonance imaging (MRI) and computed tomography (CT) scans, were viewed whenever accessible to establish an unequivocal diagnosis. In assessing whether a fracture was present, the criterion was to evaluate only acute fractures, while older fractures were not considered.

### 2.3. Algorithm

This study was conducted using the Gleamer BoneView© (Gleamer, Paris, France) algorithm. This program utilizes an algorithm based on a deep convolutional neural network built on the “Detectron 2” framework [17]. The primary objective of this algorithm is to detect and display fractures on digital radiographs. For programming and training purposes, a dataset consisting of 60,170 radiographic images was used. This dataset comprised radiographs obtained from trauma patients and was collected from 22 different healthcare facilities over several years, from 2011 to 2019. The dataset was randomized, with 70% of the data used for training, 10% for validation, and 20% for internal validation purposes [14,15]. Findings identified by the AI and categorized as “doubt” were considered to be fractures. This categorization was based on the AI recognizing the possibility of a fracture in these cases. 

### 2.4. Ethics Statement

This study was conducted in accordance with all guidelines established by the approving review board of the investigating hospital (approval code: 23-11109-BO).

### 2.5. Statistical Analysis 

This study evaluated the performance of radiology residents in fracture detection on radiographs, both with and without the assistance of an AI-powered fracture detection system. The accuracy of fracture detection was of particular interest in assessing the performance of the radiology residents. 

To evaluate the accuracy of fracture detection, several parameters were determined:

- True positive (*TP*): a fracture was detected when a fracture was present.
- False negative (*FN*): no fracture was detected when a fracture was present.
- False positive (*FP*): a fracture was detected when no fracture was present.
- True negative (*TN*): no fracture was detected when no fracture was present.
- Sensitivity: the proportion of true positive cases correctly identified as fractures by the radiology residents.
  $$\frac{TP}{TP+FN}$$
- Specificity: the proportion of true negative cases correctly identified as non-fractures by the radiology residents.
  $$\frac{TN}{TN+FP}$$
- Positive predictive value (PPV): the proportion of cases identified as fractures by the radiology residents that were confirmed as true fractures.
  $$\frac{TP}{TP+FP}$$
- Negative predictive value (NPV): the proportion of cases identified as non-fractures by the radiology residents that were confirmed as true non-fractures.
  $$\frac{TN}{TN+FN}$$

These parameters were calculated based on the comparison of the radiology residents’ interpretations with the reference standard, which was established by expert radiologists. In addition, the parameters for the performance of the AI compared to the ground truth were determined. The corresponding 95% confidence interval (95% CI) was determined for each diagnostic accuracy measurement. The analysis of the rates was conducted both with and without AI assistance, as well as a separate evaluation of the AI’s performance on its own. This comprehensive examination allows for direct comparison and understanding of the impact of AI assistance on the diagnostic accuracy of radiology residents.

Statistical analyses were conducted using GraphPad Version 10.0.0 (131) (© 1994–2023 GraphPad Software, LLC, Boston, MA, USA). A *p*-value of less than 0.05 was considered statistically significant. The Shapiro–Wilk test was used to determine the normality of the data. The data were not normally distributed. Therefore, to test the data for significant differences, the non-parametric Mann–Whitney test for non-normally distributed unpaired groups was performed. The hybrid Wilson/Brown method was used to compute the confidence interval.

## 3. Results

In this study, the accuracy of radiology residents’ findings with and without AI support, AI stand-alone performance, and the approved report in the radiology information system (RIS) were analyzed. The time required by radiology residents to report findings and their confidence in reporting were also assessed.

The sensitivity of radiology residents was significantly higher with AI support (77% [95% CI: 72–82]) than without AI support (58% [95% CI: 52–64]; *p* = 0.001). There was little difference in specificity between the two groups (77% [95% CI: 71–81] vs. 79% [95% CI: 73–84]). The PPV was 7% higher with AI support (81% [95% CI: 76–86] vs. 74% [95% CI: 67–79]) and the NPV was 13% higher with AI support (75% [95% CI: 69–80] vs. 62% [95% CI: 56–67]). The most experienced radiology resident (24.5 months; Resident 1) showed only minor improvements in diagnostic performance with AI support. For the less experienced radiology residents (4.5–18.5 months; Residents 2–4), the differences with and without AI support were markedly higher (Table 1). Therefore, the overall diagnostic performance of the radiology residents (Residents 1–4) showed a clear improvement as a result of the AI support. These differences were particularly evident in the less experienced radiology residents (4.5–18.5 months; Residents 2–4). In contrast, the most experienced radiology resident (24.5 months; Resident 1) showed almost a deterioration in diagnostic performance with AI support (Table 1).

Compared with AI alone, residents without AI support detected significantly fewer fractures (*p* < 0.0001). Substantial differences were observed in the sensitivity of residents without AI support (58% [95% CI: 52–64]) and AI stand-alone performance (93% [95% CI: 87–96]). For NPV, the results of the AI stand-alone performance were also considerably higher (62% [95% CI: 56–67] vs. 89% [95% CI: 82–94]). The difference between the two groups was low for PPV at 9% (74% [95% CI: 67–79] vs. 83% [95% CI: 77–88]). Specificity was identical in both groups on average (77% [95% CI: 71–81] vs. 77% [95% CI: 69–84]) (Table 2).

When residents were assisted by the AI, only slight differences were observed compared to AI stand-alone performance. The largest differences were seen in sensitivity at 16% (77% [95% CI: 72–82] vs. 93% [95% CI: 87–96]) and NPV at 12% (75% [95% CI: 69–80] vs. 89% [95% CI: 82–94]). Specificity (79% [95% CI: 73–84] vs. 77% [95% CI: 69–84]) and PPV (81% [95% CI: 76–86] vs. 83% [95% CI: 77–88]) showed marginal differences between the two groups (Table 2).

When comparing the approved radiological reports in the RIS, where the radiographs were initially interpreted by a radiology resident and then corrected by an experienced radiologist, with the AI stand-alone performance, only minor differences could be observed between the groups. The groups differed by less than 10% in all four diagnostic performance measurements. Specifically, the sensitivity of the AI stand-alone performance was 8% higher (93% AI vs. 85% RIS), while the specificity of AI alone was slightly lower compared to the RIS findings (82% AI vs. 89% RIS). Table 3 shows the differences between AI and approved radiological reports.

When residents were supported by the AI during reporting, they required an average of 29.6 s (standard deviation (SD) ± 19.8 s). When residents were not supported by the AI, they needed an average of 32.2 s (SD ± 20.8 s). The difference in reporting time between the two groups was significant (*p* = 0.0156). When AI support was used, residents felt more confident in making findings (1.53 ± 0.91) than when AI support was not used (1.72 ± 1.02) (*p* = 0.0013) (Table 4).

## 4. Discussion

The purpose of this study was to evaluate AI support for fracture detection in pediatric and adult trauma patients and to investigate the impact on sensitivity, specificity, and confidence of residents. 

When radiology residents received AI support, their ability to detect fractures exhibited a significant improvement in sensitivity, reaching 77% vs. 58% when they relied solely on their expertise without AI support. These results are consistent with the findings of previous studies that have reported the potential of AI in improving radiologists’ sensitivity [4,11,12,14,15,16]. For example, Nguyen et al. found that the sensitivity of experienced and young radiologists in diagnosing fractures of the appendicular skeleton in children and young adults increased from an average of 73.3% to 82.8% with the use of AI [16]. In a multi-center cross-sectional diagnostic study aimed at detecting adult appendicular skeletal fractures by emergency physicians and radiologists, Duron et al. observed a significant increase in sensitivity, with the introduction of AI support resulting in a statistically significant enhancement from 70.8% to 79.4% [14]. 

The slightly lower sensitivity in fracture detection in this study may be related to the study’s focus on radiology residents and the improvement in radiology training. In contrast, other studies have evaluated the performance of radiologists, some of whom possess many years of experience [15,16]. Furthermore, other studies often focus on a specific region of the body [11,14,16]. This study, in contrast, was designed to cover a wide range of different body regions based on the frequency of occurrence in the emergency department of the investigating hospital.

The radiology resident with the most extensive experience demonstrated only slight improvements with AI assistance, whereas the remaining residents exhibited tremendous improvements with AI support. A possible explanation for this discrepancy could be a higher level of expertise in fracture detection, resulting in no benefit from AI support. Consequently, less experienced residents may benefit more from AI support to improve their skills. Furthermore, it is conceivable that more experienced residents with greater knowledge may be less dependent on AI. Further studies with a larger and more diverse group of radiology residents would be needed to confirm these hypotheses.

In this study, AI exhibited superior sensitivity compared to all radiology residents. AI achieved a sensitivity of 93% when operating independently. The results emphasizing the high sensitivity of AI in stand-alone performance are consistent with the current literature, which frequently reports AI sensitivities exceeding 80% [8,9,14,18,19,20,21,22,23,24]. In this study, there was only a minor difference in specificity between the two groups (77% vs. 79%). This is in line with the existing literature, which also reported no significant decrease in specificity when using AI for fracture detection [14,15,16]. The performance of stand-alone AI did not exhibit a statistically significant difference from the radiological findings in RIS, where an initial assessment by a resident is followed by correction by an experienced specialist. However, there was a non-significant increase in sensitivity observed in the AI stand-alone performance, with a sensitivity of 93% compared to 85% in the approved reports (*p* = 0.2809). Conversely, the specificity in the RIS findings was higher at 83% compared to the stand-alone AI performance of 77%. 

The improvement in fracture detection with AI support underscores the potential of AI tools in radiology. Furthermore, it is important to investigate whether this increase in sensitivity and specificity of residents is associated with a significant increase in the time required for radiograph interpretation. In this study, we also recorded the reading time per case. Here, a small but significant difference in reading time was identified between the groups (*p* = 0.0156). With AI support, the mean time per case was 29.6 s (SD ± 19.8 s), whereas without AI support, it was 32.2 s (SD ± 20.8 s). Overall, the utilization of AI in the interpretation of radiographs by radiology residents leads not only to increased sensitivity but also to a reduction in interpretation time. In line with this, Guermazi et al. also observed a significant time saving along with a significantly improved sensitivity, similar to this study [15]. Duron et al. also reported a reduction in average reading time with AI support, in line with our findings [14]. One explanation for this reduction in time may be attributed to the increased confidence that residents experienced in using AI support during the evaluation process, which was also demonstrated in this study. 

Integrating AI into radiological education brings considerable advantages, such as instant feedback and a deeper understanding of radiological patterns. Nonetheless, it poses challenges, such as the possible erosion of diagnostic skills in the absence of AI and the hazard of affirming incorrect interpretations. Thoughtful planning is crucial for embedding AI into the curriculum in a way that supplements, rather than supplants, traditional teaching methods. Despite these obstacles, AI has the potential to greatly enhance the educational experience. Future studies should focus on fine-tuning the balance between AI-enhanced and traditional learning to amplify benefits and curtail disadvantages.

In summary, this study revealed an enhancement in sensitivity among radiology residents in the interpretation of radiographs of trauma patients, accompanied by a reduction in reporting time and increased confidence in their interpretations, attributable to the use of AI support during the interpretation process. The potential of educational tools in the further professional training of resident physicians has been several times [25,26,27]. AI assistance in the interpretation of radiographic images could represent an additional component in the professional advancement of resident physicians.

Several limitations should be noted. The assessment of radiographs relies on essential clinical information. Due to substantial variability in the quality and availability of clinical data, we opted not to incorporate it entirely in this study. Furthermore, the selection of regions for examination was based on the frequency of cases seen in the emergency department of the single-center institution where the study was conducted, aiming to provide a realistic representation of trauma patients. However, the distribution of cases may significantly differ in other hospital settings. Multi-center study designs could address this variability in future investigations. As captured in this study, the performance of radiology residents may vary significantly on an individual basis. Future research should include a larger cohort of radiology residents from different healthcare facilities. Additionally, a larger sample size could enable a more precise analysis of different anatomical regions and accommodate the varying complexity of fracture detection. Additionally, the retrospective nature of this study should be considered, which entails specific limitations, including the potential for biases, that could restrict the generalizability of the results to other contexts. It should also be noted that the ground truth was established by experienced radiologists rather than cross-sectional imaging. Available cross-sectional imaging was deliberately not set as an inclusion criterion, as it is typically reserved for cases with uncertain fractures and could introduce bias by over-representing challenging radiographs. Finally, future investigations could examine the performance of the same radiology residents with and without AI support, especially in the context of ongoing resident training.

## 5. Conclusions

In conclusion, this study demonstrated that AI support significantly improved fracture detection sensitivity and specificity among less experienced radiology residents. Additionally, AI reduced interpretation time, likely due to improved resident confidence. This study highlights the potential of AI in radiology to improve sensitivity and efficiency, with implications for training and interpretation.

## Figures and Tables

**Table 1:** Comparison of diagnostic performance between radiology residents without AI support and performance with support from the AI fracture detection tool. The difference is calculated based on the improvement or deterioration between the diagnostic performance without AI support and with AI support.

	Without AI Support	AI Supported	Difference
Resident 1			
Sens (95% CI)	81% (69–89)	82% (72–89)	1%
Spec (95% CI)	82% (71–90)	69% (56–79)	−13%
PPV (95% CI)	81% (69–89)	78% (68–85)	−3%
NPV (95% CI)	82% (71–90)	74% (61–84)	−8%
Resident 2			
Sens (95% CI)	48% (36–61)	70% (59–79)	22%
Spec (95% CI)	75% (64–84)	77% (64–87)	2%
PPV (95% CI)	64% (49–76)	82% (71–89)	18%
NPV (95% CI)	62% (51–72)	64% (52–75)	2%
Resident 3			
Sens (95% CI)	57% (46–68)	78% (65–86)	21%
Spec (95% CI)	78% (65–87)	89% (79–95)	11%
PPV (95% CI)	79% (66–87)	88% (77–94)	9%
NPV (95% CI)	56% (45–67)	80% (68–88)	24%
Resident 4			
Sens (95% CI)	49% (38–60)	79% (67–88)	30%
Spec (95% CI)	71% (58–81)	81% (69–89)	10%
PPV (95% CI)	69% (56–80)	79% (67–88)	10%
NPV (95% CI)	51% (40–62)	81% (69–89)	30%
Residents 1–4			
Sens (95% CI)	58% (52–64)	77% (72–82)	19%
Spec (95% CI)	77% (71–81)	79% (73–84)	2%
PPV (95% CI)	74% (67–79)	81% (76–86)	7%
NPV (95% CI)	62% (56–67)	75% (69–80)	13%

Note: Presentation of diagnostic performance measures, including sensitivity (Sens), specificity (Spec), positive predictive value (PPV), and negative predictive value (NPV) and their associated 95% confidence intervals (95% Cis).

**Table 2:** Comparison of diagnostic performance between radiology residents without AI support, with AI support, and the AI fracture detection tool.

	Residents without AI Support	Residents with AI Support	AI
Sens (95% CI)	58% (52–64)	77% (72–82)	93% (87–96)
Spec (95% CI)	77% (71–81)	79% (73–84)	77% (69–84)
PPV (95% CI)	74% (67–79)	81% (76–86)	83% (77–88)
NPV (95% CI)	62% (56–67)	75% (69–80)	89% (82–94)

Note: Presentation of diagnostic performance measures, including sensitivity (Sens), specificity (Spec), positive predictive value (PPV), and negative predictive value (NPV) and their associated 95% confidence interval (95% CIs).

**Table 3:** Comparison of diagnostic performance of initial radiological findings and the AI tool for fracture detection.

	RIS	AI	Difference
Sens (95% CI)	85% (78–90)	93% (87–96)	8%
Spec (95% CI)	83% (75–89)	77% (69–84)	−6%
PPV (95% CI)	86% (79–91)	83% (77–88)	−3%
NPV (95% CI)	82% (74–88)	89% (82–94)	7%

Note: Presentation of diagnostic performance measures, including sensitivity (Sens), specificity (Spec), positive predictive value (PPV), and negative predictive value (NPV) and their associated 95% confidence intervals (95% CIs).

**Table 4:** Comparison of resident diagnostic time and confidence with and without AI support.

	Reporting Time	Reporting Time	Confidence	Confidence
	AI Supported	without AI Support	AI Supported	without AI Support
Resident 1	45.8 s	48.6 s	1.53	1.55
Resident 2	26.6 s	25.2 s	1.64	1.52
Resident 3	23.1 s	28.9 s	1.48	2.14
Resident 4	23.1 s	26.1 s	1.36	1.59
Residents 1–4	29.6 s ± 19.8 s	32.2 s ± 20.8 s	1.53 ± 0.91	1.72 ± 1.02

## Data Availability

Data generated or analyzed during this study are available from the corresponding author by request.
